# A data-driven approach to the “Everesting” cycling challenge

**DOI:** 10.1038/s41598-023-29435-w

**Published:** 2023-02-08

**Authors:** Junhyeon Seo, Bart Raeymaekers

**Affiliations:** Department of Mechanical Engineering, Virginia Tech, Blacksburg, VA 24061 USA

**Keywords:** Physiology, Engineering, Scientific data

## Abstract

The “Everesting” challenge is a cycling activity in which a cyclist repeats a hill until accumulating an elevation gain equal to the elevation of Mount Everest in a single ride. The challenge experienced a surge in interest during the COVID-19 pandemic and the cancelation of cycling races around the world that prompted cyclists to pursue alternative, individual activities. The time to complete the Everesting challenge depends on the fitness and talent of the cyclist, but also on the length and gradient of the hill, among other parameters. Hence, preparing an Everesting attempt requires understanding the relationship between the Everesting parameters and the time to complete the challenge. We use web-scraping to compile a database of publicly available Everesting attempts, and we quantify and rank the parameters that determine the time to complete the challenge. We also use unsupervised machine learning algorithms to segment cyclists into distinct groups according to their characteristics and performance. We conclude that the power per unit body mass of the cyclist and the tradeoff between the gradient of the hill and the distance are the most important considerations when attempting the Everesting challenge. As such, elite cyclists best select a hill with gradient > 12%, whereas amateur and recreational cyclists best select a hill with gradient < 10% to minimize the time to complete the Everesting challenge.

## Introduction

“Everesting” is a cycling activity in which a rider repeatedly ascends and descends (part of) a hill until accumulating 8848 m of elevation gain—the elevation of Mount Everest above sea level—in one ride^1^. The first successful Everesting attempt was recorded in 1994 by George Mallory, grandson of famed Everest mountaineer George Mallory, who completed eight consecutive ascents of Mount Donna Buang in Australia^2^. However, it was not until 2014 before Everesting rules were formalized^3^ and the everesting.cc website started to catalog Everesting results^4^. While initially only a few tens or hundreds of cyclists attempted this new challenge, data shows that more than 1000 cyclists completed it in 2019, and almost 10,000 in 2020^5^. This surge in interest was fueled in part by the COVID-19 pandemic and the cancelation of cycling races around the world that caused cyclists to pursue individual cycling activities as an alternative to races. The Everesting challenge also drew media attention^6–10^ because several (former) pro-cyclists—including a Tour de France champion^6^—completed the challenge in new record times, which created awareness and spurred interest with the general public.

Everesting works as follows. Cyclists select a hill and record their attempt on Strava^11^, a well-known social media application for GPS-tracking of human exercise and in particular cycling. Subsequently, they submit the Strava data to the everesting.cc website, where it is posted after reviewing that the number of repetitions of the selected Strava segment meets the required number to reach 8848 m of elevation gain. Public data of each attempt on the everesting.cc website includes cyclist information (screen name, age, gender, number of completed Everesting attempts), course information (Strava segment, location, average gradient of the hill, number of hill repeats, distance, and time to complete the Everesting challenge), and environmental information (temperature). Only limited information about cyclist performance is available. For instance, the everesting.cc data allows calculating the average speed, which derives from the cyclist’s performance but also depends on the gradient of the hill, among other parameters. Furthermore, the time to complete the Everesting challenge includes both time ascending and descending, but only the former is of interest in terms of measuring cyclist performance. Traditionally, the (average) power per unit body mass (Watt/kg) quantifies cyclist performance, as it relates to fitness and talent of the cyclist, independent of the hill or environment used for the Everesting challenge^12^. However, such data is often difficult to determine or simply unavailable, because most recreational cyclists do not use a power meter on their bicycle and pro- or amateur-level cyclists do not always share power information publicly, as it could provide a competitive advantage to others. The weight of each cyclist (on the day of the Everesting attempt) is also generally not available.

The central question of the Everesting challenge revolves around quantifying and ranking the relative importance of the Everesting parameters that determine the time to complete the challenge, and identifying metrics that are common to successfully complete the challenge, or even compete for the world record. Few reports exist in the open literature that attempt to address this question. Cesanelli et al. analyzed the parameters necessary to optimize a training protocol for an ultra-endurance performance such as the Everesting challenge, and they concluded that a 10-week training protocol can be sufficient for an elite athlete to complete the challenge, thus emphasizing the importance of fitness^13^. Most recently, Swinnen et al. studied the biomechanics and energetics of up- and downhill cycling to determine the characteristics of the optimal hill in the context of the Everesting challenge^14^. Their theoretical analysis indicated that a 2–3 km hill close to sea level, with a slope of 12–20% leads to the fastest time to complete the Everesting challenge. Additionally, they concluded that 24 hill repeats are optimal to minimize time to complete the challenge, based on the critical power concept^15^. Despite media attention^2,4,6–10,16,17^ and interest from cyclists worldwide, no reports exist in the open literature that methodically document and analyze the Everesting challenge based on experimental data.

However, analyzing aggregate data of past Everesting attempts can shed light on the parameters that determine the time to complete the Everesting challenge and, consequently, provide insight into selecting parameters to compete for the Everesting world record in the case of a pro- or amateur-level cyclist, or to simply complete the challenge for a recreational cyclist. Thus, the objective of this work is to quantify and rank the relative importance of the parameters that determine the time to complete the Everesting challenge, based solely on publicly available data of previous attempts. We also segment cyclists into distinct groups to evaluate whether different parameters and metrics are common to Everesting success for those groups. Finally, we formulate recommendations to select parameters for an Everesting attempt. This knowledge sheds light on the complex interplay between selecting parameters and successfully completing the Everesting challenge. Beyond the Everesting challenge, this work illustrates that elementary data analysis can reveal useful information hidden within a real-life, imperfect, publicly available dataset.

## Methods

We used webscraper.io to extract the publicly available data of 22,747 completed Everesting attempts from the everesting.cc website (on 08/24/22). This dataset includes cyclist screen name, age, gender, number of completed Everesting challenges, and the Strava segment, average gradient, distance, total time, number of hill repeats, and temperature at the start of the Everesting attempt. While other parameters such as the relative humidity and bicycle weight may also affect the time to complete the Everesting challenge, such information was not available on the Everesting.cc website.

To only consider successful attempts of the Everesting challenge in our analysis, we filtered the data based on multiple integrity checks. First, we disregarded all attempts that did not meet the Everesting rules, i.e., those that did not reach 8848 m of elevation gain, those that lasted longer than 2 days (because it must be a continuous attempt and it is unlikely a cyclist could ride for more than 48 h without sleep), and those that reported zero hill repeats. We also eliminated attempts that did not report a complete set of parameters (cyclist, course, environment). Furthermore, we chose to only consider real-world Everesting attempts and, thus, we removed all “virtual rides” completed on a stationary bicycle in combination with a platform like Zwift^18,19^. We did not consider attempts that are physically unlikely or even impossible. Therefore, we removed attempts of cyclists younger than 16 years old because it is unclear whether this data is reliable. Additionally, we removed attempts where the reported distance deviated more than 10% from the theoretical distance of the Everesting attempt based on the average gradient of the hill and the reported number of hill repeats. Finally, we removed attempts with an average speed that exceeds 40 km/h, since it is unlikely that any rider could sustain such as speed without, e.g. drafting behind or holding onto a motor vehicle or using an e-bike, while conquering 8848 m of elevation gain. After applying these filters, we retained a dataset with 2561 realistic records of completed Everesting challenges (2403 male, 158 female).

The time to complete the Everesting challenge is the target attribute (output), whereas the power per unit body mass, distance, age, number of hill repeats, temperature, and gradient are the input attributes. We emphasize that the time to complete the Everesting challenge reflects the total time to complete the actual elevation gain each cyclist reports, which is at least 8848 m but sometimes exceeds the minimum value (see Table 1 for descriptive statistics). Distance and gradient are not independent of each other because the distance required to accumulate 8848 m of elevation gain increases with decreasing gradient. Furthermore, in the absence of power information for each Everesting attempt, we estimate the average power* P* per unit body mass *m*^20^ based on the actual elevation gain and time to complete the attempt, as recorded on the everesting.cc website, because intuitively the fitness and talent of the cyclist have a substantial effect on the time to complete the Everesting challenge. The total energy expended to overcome *h*_*tot*_ elevation gain is *E*_*tot*_ = *mgh*_*tot*_, which is the change in potential energy, with *g* the gravitational acceleration. In reality, a cyclist also has to overcome energy losses related to rolling resistance, aerodynamic drag, and friction forces in the bearings of the bicycle^21^, which we neglect in this simplified estimate because while riding uphill at low speed, they are small compared to the change in potential energy. The ratio of the energy expended and the time to ascend *t*_*asc*_ approximates the average power during the Everesting attempt, i.e., *E*_*tot*_/*t*_*asc*_ = *mgh*_*tot*_/*t*_*asc*_. Consequently, the average power per unit body mass *P*/*m* = *E*_*tot*_/*mt*_*asc*_ = *gh*_*tot*_/*t*_*asc*_. However, *t*_*asc*_ is unknown because instead, cyclists report the total time to complete the Everesting challenge *t*_*tot*_ = *t*_*asc*_ + *t*_*des*_, i.e., the sum of the time to ascend *t*_*asc*_ and descend *t*_*des*_, with *t*_*asc*_ = *αt*_*tot*_, and *α* < 1 depending on cyclist, hill, bicycle, environment, and other parameters. Here, we assume that *α* is constant for all cyclists, and we set *α* = 1 for simplicity since *t*_*asc*_ >  > *t*_*des*_ and, thus, *t*_*asc*_ ≈ *t*_*tot*_. In reality, *α* ≈ 7/8 based on the analysis of several Everesting world record attempts for which detailed information is available on Strava^17^; however, the choice of *α* does not affect later discussions. A fundamental shortcoming of the power per unit body mass estimate is that it necessarily depends on the time to complete the Everesting challenge, which is the target attribute, rather than representing an independent measurement. However, in the absence of the latter, we chose to include the estimate because the actual elevation gain in each Everesting attempt is sufficiently diverse between attempts and, intuitively, it plays an important role.

**Table 1:** Descriptive statistics of the Everesting dataset.

	Gradient [%]	Distance [km]	Elevation [m]	Time [hours]	Hill repeats	Age [years]	Temperature [°C]
Maximum	17.20	587.50	29,146.00	47.99	1001.00	70.00	34.00
Minimum	3.58	105.74	8849.00	7.67	2.00	17.00	− 7.00
Average, *µ*	7.57	261.05	9234.67	18.73	63.24	38.54	16.24
Median	7.25	256.05	9017.00	18.00	45.00	38.00	16.00
Standard deviation, *σ*	1.94	65.56	857.79	5.10	63.92	10.75	5.76

We first determine the normalized linear correlation coefficient between each input attribute and the target attribute to quantify and rank the relative contribution of each input attribute to the target attribute, thus identifying the most important parameters to consider in preparing for the Everesting challenge^22^. Then, we perform cluster analysis and segment the dataset into homogeneous clusters of distinct cyclist types, based on unsupervised machine learning algorithms including *k*-means, *k*-medoids, density methods (DBSCAN), spectral methods, and Gaussian mixture methods (GMM)^23,24^ with the Expectation–Maximization (EM) algorithm initialized with the *k*-means algorithm. We consider all attributes in the cluster analysis, including distance, gradient, power per unit body mass, age, number of hill repeats, temperature, and time to complete the Everesting challenge, and we evaluate the outcomes as a function of the number of clusters using internal and external metrics.

## Results and discussion

Table 1 presents the descriptive statistics of the Everesting dataset, which comprises 2561 records (see “Methods” section). We emphasize that specific Everesting parameters relate to each other; for instance, the maximum gradient of 17.20% corresponds to the shortest distance (approximately 105.74 km) and vice versa. Furthermore, as expected, most cyclists attempt the Everesting challenge when the outdoor temperature is mild 16.24 ± 5.76 °C. Roads with steep gradients (> 10%) are not available everywhere and, thus, cyclists may be restricted to selecting a hill with moderate incline 7.57 ± 1.94% for their attempt. The range of the number of hill repeats is between 2 and 1001.00, even though the majority of the data points is within 63.24 ± 63.92.

**Figure 1:**
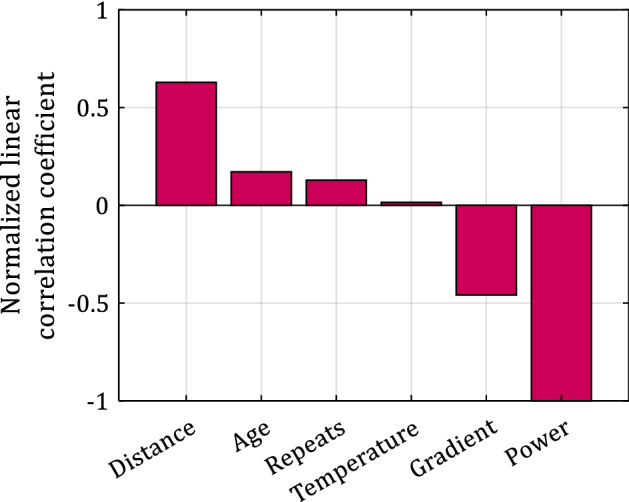
Normalized linear correlation coefficient between each Everesting (input) parameter and time (target attribute) to complete the Everesting challenge, showing that power, distance, and gradient are the main factors that determine time.

Figure 1 shows the normalized linear correlation coefficient between each Everesting parameter (input attribute) and the time to complete the Everesting challenge (target attribute), ranked in descending order. The absolute value of the correlation coefficient quantifies the relative contribution of each input attribute to the target attribute, and the sign indicates a positive or negative relationship. The effect of temperature, age, and number of hill repeats on the time to complete the Everesting challenge is smaller than that of the distance, the gradient of the hill, and power per unit body mass (see “Methods” section for power/unit mass estimation). Evidently, the correlation coefficient of the latter is − 1 since the estimate of the power per unit mass is proportional to *h*_*tot*_/*t*_*tot*_. Surprisingly, the number of hill repeats, which determines the intervals between effort (ascent) and recovery (descent) does not strongly correlate with the total time to complete the Everesting challenge. This observation contradicts the optimal number of hill repeats (24) theoretically derived by Swinnen et al.^14^. Decreasing the length of the ascent allows more frequent yet shorter recovery periods during the descent, but also requires more frequent turning at the bottom and top of the hill. The limited correlation with the time to complete the Everesting challenge indicates that the time gained by recovering more frequently during the attempt may be lost by the time needed to turn and accelerate at the bottom and top of the hill.

The correlation coefficient shows that the power per unit body mass is the most important input attribute, i.e., increasing power decreases the time to complete the Everesting challenge, which is intuitive because increasing power increases propulsion. This input attribute is dependent on a cyclist’s fitness and talent, as well as their body mass. Also, the total distance and the gradient of the hill are the two next most important input attributes, and they are partially related to each other through the number of hill repeats. The tradeoff between total distance and gradient of the hill is at the heart of the Everesting challenge. The time to complete the Everesting challenge increases with increasing distance and decreases with increasing gradient because a rider gains more elevation per unit distance. However, the average speed also decreases with increasing gradient and constant power. Reports in the media anecdotally suggest that pro-level riders, who are talented, trained, and can output a high power per unit body mass for a long time, prefer the gradient to be as steep as possible to minimize the time to complete the Everesting challenge^7,8,16,17^, which is substantiated by our dataset. For instance, the top-10 record times of the Everesting challenge (on 08/24/22) were all achieved on a hill with gradient > 10%, 8 out of 10 with gradient > 13%, and 4 out of 10 with gradient > 15%, compared to the average gradient of 7.57% calculated based on the entire dataset (see Table 1).

**Figure 2:**
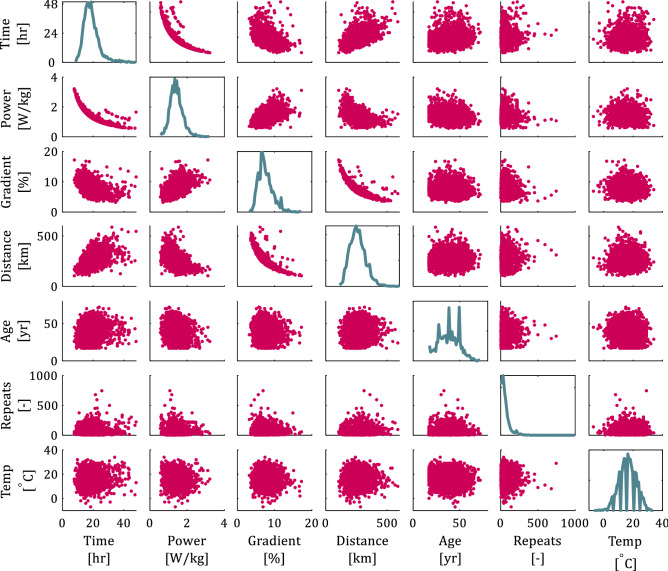
Pairwise relationship between input and target attributes in matrix format with the probability density function of each attribute along the main diagonal.

**Figure 3:**
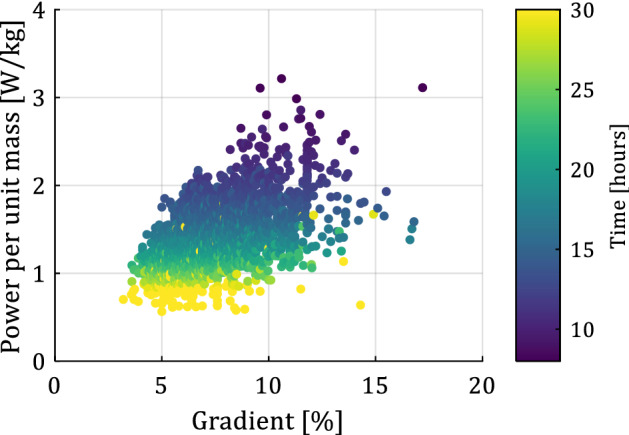
Estimated power per unit body mass as a function of the gradient, with the color of each datapoint indicating the total time (colorbar), illustrating that cyclists who can output high power per unit mass preferentially select a steep gradient.

Figure 2 shows the pairwise relationship between all input attributes and time (target attribute) in matrix format. The main diagonal shows the probability density function of each attribute. Note that even though the results of Fig. 2 show the entire dataset, the results are similar when separating male and female cyclists. We observe that the time to complete the Everesting challenge decreases with increasing gradient and increasing power, as expected from Fig. 1. All attempts that took under 10 h occurred on a hill with an average gradient > 7%. Figure 3 shows the estimated power per unit body mass versus the gradient, and the color of each datapoint represents the time to complete the Everesting challenge (see colorbar), thus illustrating that talented or well-trained cyclists preferentially select hills with steep gradients^7,8,16,17^ to capitalize on their talent and fitness, as opposed to recreational cyclists who cannot sustain such a demanding physical effort and, therefore, select a less steep hill. Hence, a cyclist’s ability to ascend steep hills fast (high power per unit body mass), rather than simply selecting a hill with steep gradient, likely drives the time to complete the Everesting challenge. Selecting a gradient below 7% most often results in attempts that exceed 20 h, driven by the increased distance of the ride (see Fig. 2). Furthermore, no unique relationship between gradient and distance exists, and the variation of the data for constant gradient or constant distance in Fig. 2 is due to the number of hill repeats, which may differ for each individual attempt as a result of selecting different hills.

**Figure 4:**
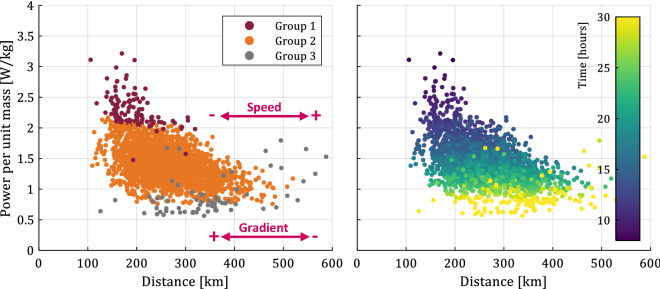
Estimated power per unit body mass versus distance, indicating different clusters of cyclists (groups 1, 2, and 3), and indicating the time to complete the Everesting challenge.

We perform cluster analysis considering all input and target attributes to segment the dataset into distinct cyclist types using unsupervised machine learning algorithms. Figure 4 shows the estimated power per unit body mass versus the distance, where we indicate the direction of increasing/decreasing gradient and speed with arrows. Each datapoint clusters into one of three distinct cyclist types: groups 1 (Chicago maroon), 2 (Burnt orange), and 3 (Hokie stone), using a Gaussian mixture model (GMM, see “Methods” section), which we determine to work better than *k*-means, *k*-medoids, density (DBSCAN), and spectral methods based on internal and external metrics. We also show the datapoints colored according to time (see colorbar) to assist with the interpretation of the different groups. Figure 4 shows that group 1 (Chicago maroon) likely represents talented, highly-trained cyclists, characterized by a high power per unit body mass. These cyclists have the ability to select a steep hill because they are able to maintain high power output for a long time, and this results in a fast time to complete the Everesting challenge, and compete for the world record. In contrast, group 3 (Hokie stone) likely represents recreational cyclists who generally select a hill with a shallow gradient because they cannot maintain high power output and, as a result, their total time to finish the challenge exceeds 20–25 h. In between groups 1 and 3, we define group 2 (Burnt orange) as a group of well-trained amateur cyclists who complete the Everesting challenge in 15–25 h, yet in general select a hill with a gradient that is shallower (4–10%) than the elite cyclists of group 1.

**Figure 5:**
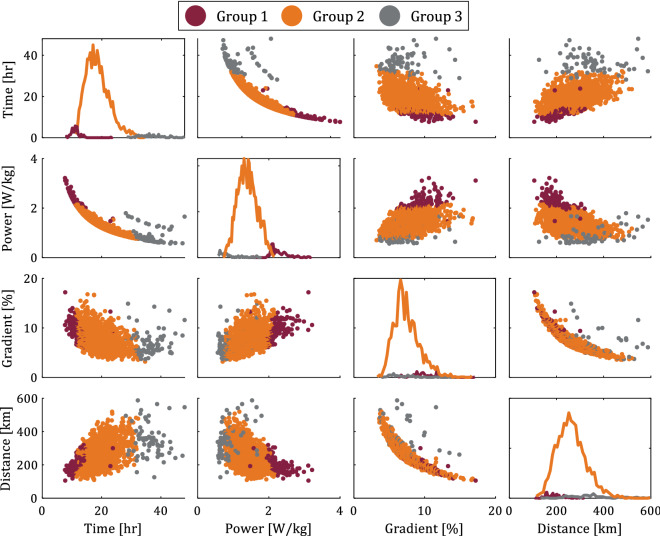
Pairwise relationship between input and target attributes in matrix format with the probability density function of each attribute along the main diagonal, and indicating different clusters of cyclists (groups 1, 2, and 3) based on a Gaussian mixture model.

Figure 5 shows the pairwise relationship between the distance, gradient, power and time (target attribute) in matrix format, similar to Fig. 2 but only showing the three input attributes that most substantially affect the target attribute based on Fig. 1. Each of these attributes approximately follows a Gaussian distribution (see main diagonal in Fig. 2) and, thus, 68% of the datapoints of each attribute are confined within one standard deviation of the arithmetic mean (see Table 1). The main diagonal shows the probability density function of each attribute, and we color each datapoint according to the three distinct cyclist types of Fig. 4; groups 1 (Chicago maroon), 2 (Burnt orange), and 3 (Hokie stone). From Fig. 5 we observe the relationship between different Everesting parameters, presented as a function of the distinct cyclist types, thus further substantiating the intuitive interpretation of these different cyclist types.

Even though not explicitly shown in Figs. 4 and 5, we determine that separating female and male cyclists yields similar results and interpretations, but it is important to emphasize that the dataset for female cyclists is an order of magnitude smaller than that of male cyclists. We also underscore the limitations of estimating the power per unit body mass in this work, which is solely based on the energy to overcome the actual elevation gain in the total time to complete the Everesting challenge, rather than based on a measurement using a power meter. Hence, it neglects energy losses from rolling resistance, aerodynamic drag, and friction forces in the bearings of the bicycle, which are small compared to the power required to overcome the elevation gain^25^. Consequently, we underestimate the power per unit body mass compared to the values documented in the media for Everesting world record attempts, which originate from power meter measurements during specific world record attempts. For instance, Sean Gardner averaged 4.73 Watt/kg on the ascents during his Everesting world record^17^, whereas Keegan Swenson averaged 3.59 Watt/kg during his entire attempt^26^. For comparison, the power per unit body mass estimate for Keegan Swenson in this paper is 3.14 Watt/kg, i.e., a difference of approximately 13%. Our estimate could be improved by considering the time ascending only, if that data would be available, instead of considering the total time. Accounting for energy losses related to aerodynamic drag, rolling resistance, and friction forces in the bearings of the bicycle would also improve the power estimate, but also requires more information. Availability of power meter data, if correctly calibrated, in combination with the weight of the cyclist, would yield the exact information.

We performed cluster analysis with different unsupervised machine learning algorithms to segment cyclists into homogeneous groups, based on their Everesting performance. Each algorithm yields a solution and the number of clusters is specified a priori. Hence, the interpretation of the results is important to select and interpret a relevant solution. We attempted *k*-means and *k*-medoids and selected between 2 and 5 clusters to segment the data, and we used internal and external metrics to evaluate the quality of the different clusters, such as shadow plots. Even though the shadow plots showed little overlap between adjacent clusters, it was not intuitive to recognize and associate different types of cyclists with the resulting clusters. We also attempted using spectral methods and density methods (DBSCAN) with limited success, likely because the datapoints are densely packed. The Gaussian mixture models (GMM) were most successful in segmenting the cyclist data into homogeneous groups, because the clustering was repeatable and driven by the input attributes that show the highest correlation coefficient with the target attribute. As a result, the algorithm segments the data into clusters based on distinct power levels, which is one of the most important parameters to distinguish between cyclists’ Everesting attempts.

## Conclusions

Based on our analysis of the aggregate dataset of completed Everesting challenges, we conclude that the power per unit body mass and the tradeoff between gradient versus distance are the primary drivers of the time to complete an Everesting challenge. The ability of a cyclist to produce a high power per unit body mass allows them to select a steep hill for the Everesting challenge and, in turn, reduce the distance and time required to conquer 8848 m of elevation gain. Surprisingly, the number of hill repeats seems unimportant with respect to the time to complete the Everesting challenge, which contrasts theoretical analysis by others. Reducing the length of the segment increases the frequency but decreases the duration of the recovery periods throughout the ride, indicating that the increased frequency of recovery is offset by time loss during turning at the bottom and top of the hill.

We recommend elite cyclists, who can sustain a high average power per unit body mass (2.5–3.0 Watt/kg, using the estimation method based on potential energy we used in this work), to select a steep hill (gradient > 12%) for their Everesting attempt, and minimize the distance and time to complete the challenge. Additionally, we recommend amateur and recreational cyclists, who cannot sustain a high average power per unit body mass, to select a hill with gradient < 10% for their Everesting attempt, to find a balance between power, gradient, and distance.

## Data Availability

All data is available upon request from the corresponding author.
